# Small Intestine Length Measurement Using 3D CT Volumetry and in Vivo Laparoscopic Measurement Using Pre-marked Graspers: A Comparative Study

**DOI:** 10.1007/s11695-025-08446-8

**Published:** 2026-01-14

**Authors:** Ahmed Abdelsalam, Ahmed Ghobashy, Ahmed Abdellatif, Tamer Elholiby, Ahmed Haitham, Ahmed Khaled, Ahmed Refaat

**Affiliations:** Cairo University, Giza, Egypt

**Keywords:** 3D CT volumetry, Bariatric surgery, Pre-marked graspers, TBL

## Abstract

**Introduction:**

The method for assessing the bowel length is debatable but crucial in bariatric surgeries, especially revisional ones. It may be linked to improved weight loss and reducing nutritional deficiencies; however, it can be time-consuming, increasing the likelihood of complications during and after the surgery. 3D CT volumetry could offer a non-invasive, time-saving alternative for accurate Total Bowel Length (TBL) measurement, potentially reducing operative time and associated risks.

**Objective:**

This study compares small bowel length using 3D CT volumetry versus in vivo laparoscopic measurement with pre-marked graspers, and assesses the time consumed intra-operatively for TBL measurement in cases of revisional bariatric surgery.

**Methods:**

This cross-sectional study included 34 bariatric surgery candidates undergoing revisional or conversion bariatric surgery requiring bowel length estimation. Surgeries included Roux-en-Y gastric bypass (RYGB), single anastomosis duodeno-ileal bypass with sleeve gastrectomy (SADI-S), one-anastomosis gastric bypass (OAGB), banded Roux-en-Y gastric bypass (banded RYGB), and distalization. Pre-operatively, 3D CT volumetry-based bowel length measurements were performed and interpreted by the same radiologist, and compared with intraoperative bowel measurements, which were estimated in vivo laparoscopically using pre-marked graspers. A single surgeon did all the surgical procedures.

**Results:**

The participants had an average age of 42.0 years and a mean BMI of 40.4 ± 7.6 kg/m². The cohort was predominantly female (73.5%). The bowel length estimation took an average of 19 ± 4 min intra-operatively. A comparison of radiographic and intraoperative measurements showed no significant differences for TBL (*P* = 0.264). The intraclass correlation (ICC) for radiographic and intraoperative measurements showed that radiographic TBL shows acceptable consistency (*P* = 0.007).

**Conclusion:**

3D CT volumetry can be considered a reliable and safe method for TBL assessment. It aids in accurate preoperative planning, reduces operative time, and avoids the risks encountered during intraoperative bowel measurements in bariatric surgery.

**Key Points:**

• * An ideal bowel length should achieve effective weight loss while minimizing nutritional deficiencies.*

• * Estimation of total bowel length using 3D CT volumetry shows reliable results compared to intraoperative measurements.*

• * Estimation of total bowel length using 3D CT volumetry minimizes operative time.*

**Supplementary Information:**

The online version contains supplementary material available at 10.1007/s11695-025-08446-8.

## Introduction

Over the past few decades, bariatric surgeries have evolved and gained popularity as one of the most effective weight management methods. Roux-en-Y gastric bypass (RYGB), one-anastomosis gastric bypass (OAGB), and single-anastomosis duodenoileal bypass with sleeve gastrectomy (SADI-s) are among the most frequently performed bariatric procedures nowadays, offering sustainable and comparable results regarding weight reduction and nutritional deficiencies [1, 2].

Determining optimal bowel length during bypass procedures remains an area of research, with no standardized guidelines. An ideal length should achieve effective weight loss while minimizing nutritional deficiencies [3].

Failure to accurately measure bowel length can result in suboptimal clinical response and is linked to a greater occurrence of complications after surgery [3].

To determine the optimal lengths of the alimentary and biliopancreatic limbs, total bowel length (TBL) should first be assessed [4].

For many years, researchers have been seeking a reliable and time-efficient method to measure small bowel length accurately. The traditional approach employs a measuring tape along the anti-mesenteric border without applying any tension [5].

This method has proved its reliability in open surgeries; however, it is less practical for laparoscopic procedures, making it challenging for bariatric surgeries, which are now almost exclusively performed laparoscopically [6].

The application of this method in laparoscopic surgeries is impacted by numerous factors, such as sufficient exposure, the amount of stretch applied during measurement, the tool utilized for measurement, and the surgeon conducting the procedure. This makes it more time-consuming, in addition to raising the likelihood of small bowel injury [7].

Non-invasive, accurate measurement of small bowel length is a promising approach, using imaging modalities to pre-operatively measure it [8].

Computed tomography (CT) has proven efficacy. Radiographic length measurements show a strong relationship with intraoperative assessments, especially when the total lengths of the bowel are reduced, such as in short bowel cases [9].

Previous research within bariatric surgery has explored a wide range of methods for assessing small intestine length, including radiographic and CT-based imaging techniques [7–11], conventional radiography [12], and advanced 3D reconstruction systems [13–15] as well as laparoscopic measurement approaches using marked instruments and hand-over-hand techniques [16–18], collectively highlighting both the opportunities and persistent challenges in achieving accurate, reproducible bowel length measurements.

Unfortunately, measurements obtained via imaging can be confounded by several anatomic, physiological, and geometric factors [8].

To the best of our knowledge, no study has evaluated the efficacy of CT in measuring small bowel length prior to revisional bariatric surgeries to determine its reliability in measuring TBL.

Thus, this study aims to compare small bowel length measured using three-dimensional (3D) CT volumetry with in vivo laparoscopic measurement using pre-marked graspers, as well as assessment of time consumed intra-operatively for TBL measurement in cases of revisional bariatric surgery.

## Patients and Methods

This is a cross-sectional analytic study that included 34 patients undergoing bariatric surgery at the General and Laparoscopic Surgery Department of a tertiary university-based hospital from Dec 1st ,2024, to Jun 1st, 2025. It includes individuals aged between 18 and 70 years who underwent conversional or revisional bariatric surgery that required bowel length estimation, including RYGB, OAGB, and SADI-s. We excluded those who had prior intestinal surgeries or those with difficulty in intraoperative bowel measurement, i.e., extensive adhesions.

### Sample Size

The size of the sample was determined using the Clinicalc sample size calculator for analytic studies, using an alpha error of 0.05 and a study power of 0.80, and a confidence interval of 95%. According to the literature, CT volumetry had superior accuracy (2.1 ± 3.7%) over laparoscopic measurement (8.7 ± 13.7%) [10]. The sample size for studying small intestine length measurement using CT volumetry and in vivo laparoscopic measurement is 30 patients.

### Sampling Technique

A convenient group of patients meeting the specified inclusion and exclusion criteria was selected for the study until the predetermined total sample size was achieved.

### Ethical Considerations

The institutional Ethical Research Committee approved the study protocol, and the study was registered on ClinicalTrials.gov. Participants received a clear explanation of the procedure and objectives of the study. Prior to enrollment, written informed consent was obtained from participants, detailing the benefits and drawbacks of the procedure. Participation was optional, and participants had the right to withdraw whenever they chose. Following the principles of the Declaration of Helsinki, all procedures for data collection, input, and analysis were carried out with confidentiality.

### Preoperative Assessment

The participants underwent detailed history-taking regarding anthropometric measures, chronic diseases, and previous surgeries. After assessing each patient clinically through laboratory and radiographic investigations, the proper revisional or conversion bariatric surgery was selected.

A preoperative 3D gastric volumetry CT scan was performed with TBL estimation. The device used was a Toshiba Aquilin^®^ 16-slice, 7.5 MHU X-ray Tube CT scanner with dual 18-inch Flat-Panel LCD Monitors. The Patient Load Capacity was 200 Kg. Gastrografin was used as an oral contrast to delineate the gastrointestinal tract (GIT).

For patients who had undergone prior bariatric surgery, a low-dose scanning technique was employed: 80–100 kVp, 70–90 mAs, and an oral diluted Gastrografin 3% was used. For those who underwent sleeve gastrectomy, 500 ml of diluted Gastrografin was used, while 1.5 L was used for different types of bypass surgeries, 2 h before imaging, with the administration of an additional 200 ml of Gastrografin immediately before imaging. This protocol was not used in patients who had undergone recent surgery within 3 months. The 3D CT scan is not a routine practice in de novo cases at our institute; it is used only in revisional/conversional bariatric surgeries.

**Fig. 1:**
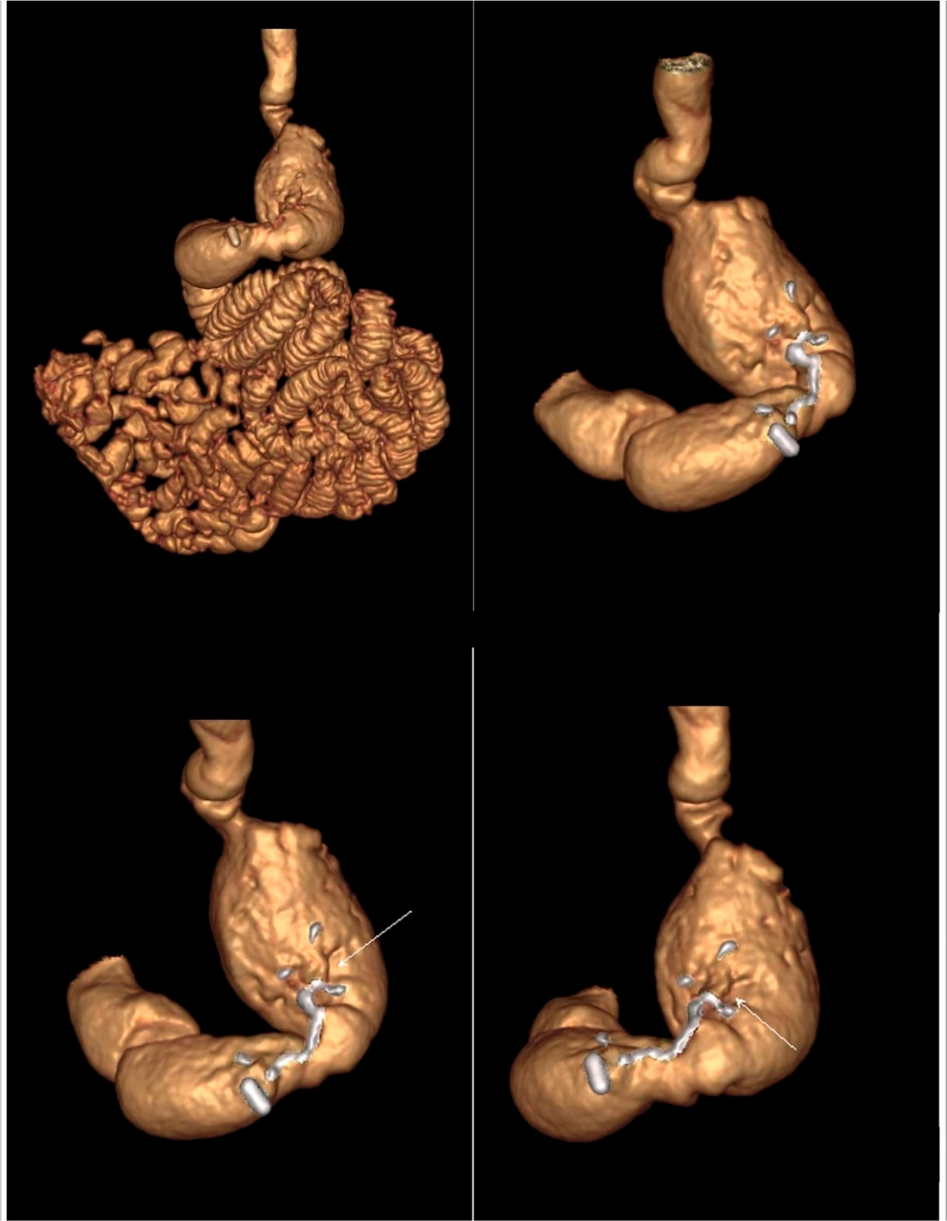
CT 3D volumetry showing a patient post-laparoscopic sleeve gastrectomy (LSG) as a primary surgery

**Fig. 2:**
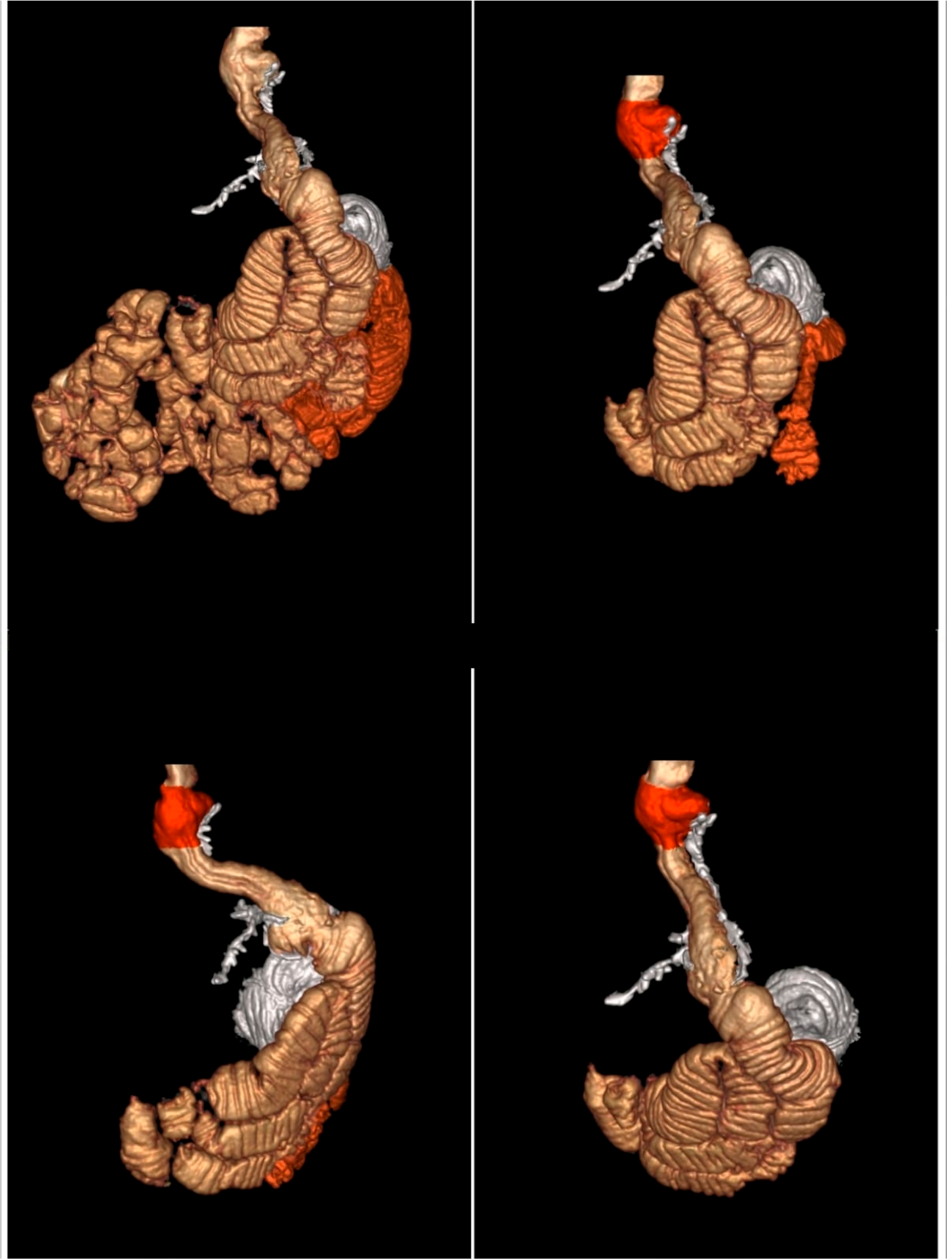
CT 3D volumetry showing a patient post-laparoscopic sleeve gastrectomy (LSG) and Roux-en-Y gastric bypass (RYGB) as primary surgeries, with a sliding hiatus hernia

For imaging processing, Virtual Reality (VR) rendering, navigation, and measurements were obtained using the GE AW Workstation **(**Figs. 1 and 2**)**.

### Operative Details

**Fig. 3:**
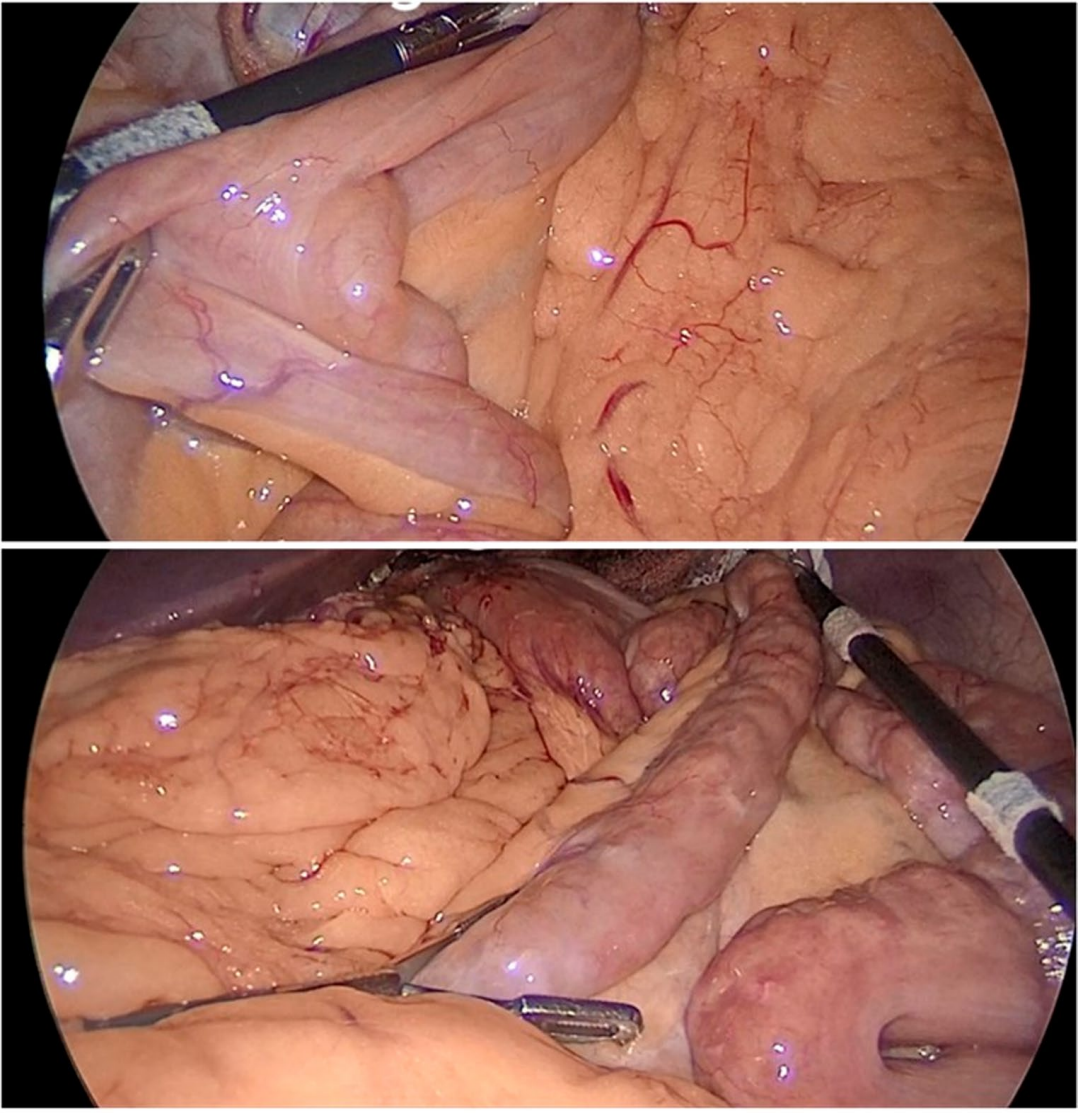
Estimating bowel length using pre-marked graspers in one of our study cases

The appropriate type of bariatric surgery was chosen according to each participant’s condition. After inducing pneumoperitoneum and placing laparoscopic ports, the transverse colon was retracted to identify the duodeno-jejunal junction (DJ). We used our marked graspers at the 5 cm (2 inches) mark to measure the small bowel length with minimal traction until the ileocecal junction was reached. The measurements were recorded and compared to those anticipated by 3D CT **(**Fig. 3**)**.

The study included participants conducted by a single bariatric surgeon, and a single radiologist interpreted their imaging. To minimize bias, the operating surgeon was blinded to the estimated bowel length reported on the CT during surgery for more accurate results.

### Postoperative Assessment

Post-operatively, patients stayed in the hospital for an average of 24 h for follow-up on vital signs and feeding regimens.

The accuracy of CT volumetry in measuring small bowel length compared to laparoscopic measurement and operative time in relation to counting the whole small bowel length was analyzed.

#### Statistical Methods

Data were entered using SPSS (Statistical Package for Social Science) version 27.0 (IBM^®^, SPSS, USA). Categorical data were presented in frequencies and percentages, while numerical data were presented in mean, standard deviation (SD), minimum, and maximum. The paired comparison was performed using the Wilcoxon signed-rank test. The agreement between CT volumetry measurements and intraoperative measurements was assessed using intraclass correlation reliability testing and visualized in a Bland-Altman plot. Any *P*-value < 0.05 was considered significant.

## Results

**Fig. 4:**
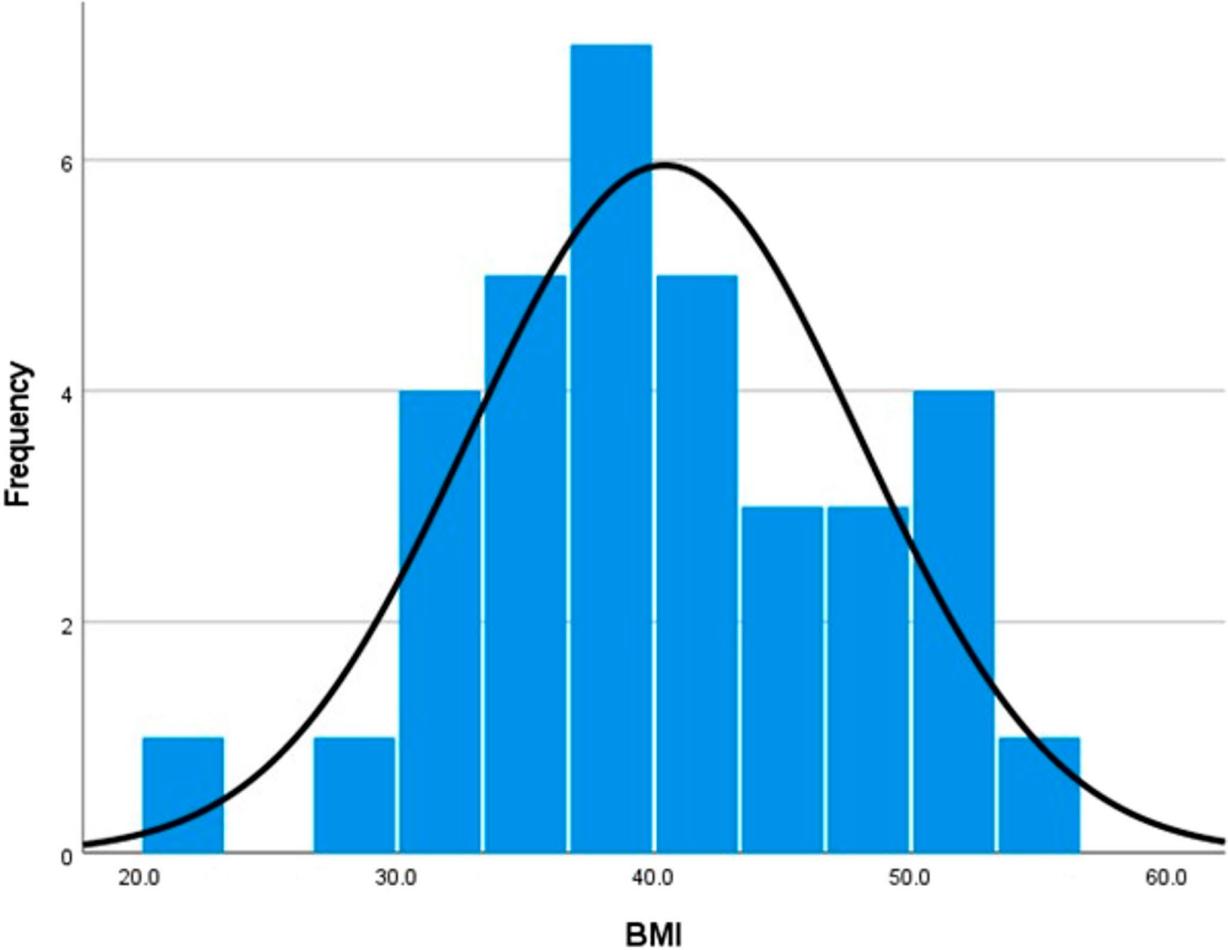
A histogram showing BMI distribution among the included patients

This study included 34 patients who met the inclusion criteria and were indicated for bariatric surgery requiring bowel length estimation. The average age of the study cohort was 42.0 years (SD ± 11.0), ranging from 17 to 59 years. Participants had an average weight of 111.6 kg (SD ± 24.2), ranging from 58.5 kg to 158.7 kg, and a mean height of 167.0 cm (SD ± 8.4), ranging from 152 cm to 188 cm. The average body mass index (BMI) was 40.4 (SD ± 7.6), with a range of 21 to 55.2. Regarding gender distribution, 26.5% (*n* = 9) of the participants were males, and 73.5% (*n* = 25) were females, reflecting a predominantly female cohort (Table 1) (Fig. 4).

**Table 1:** Anthropometric measurements among study participants

		Mean ± SD	Min, Max
Age (years)		42.0 ± 11.0	17, 59
Weight (Kg)		111.6 ± 24.2	58.5, 158.7
Height (cm)		167.0 ± 8.4	152, 188
BMI		40.4 ± 7.6	21, 55.2
		**Count**	**%**
Gender			
Male		9	26.5%
Female		25	73.5%

*BMI *body mass index,* SD *standard deviation

Among primary surgeries, laparoscopic sleeve gastrectomy (LSG) was the most frequent (47.1%), followed by vertical banded gastroplasty (VBG) at 26.4%. Other procedures, such as butterfly gastroplasty, RYGB, and various combinations of surgeries, accounted for smaller percentages (Table 2).

**Table 2:** Distribution of primary surgeries among study participants

	Types of surgeries	Count	%
Primary Surgery	LSG	16	47.1%
	VBG	9	26.4%
	Butterfly Gastroplasty	2	5.9%
	RYGB	2	5.9%
	Banded sleeve gastrectomy	1	2.9%
	LSG + RYGB	1	2.9%
	Open VBG + Open RYGB	1	2.9%
	Open VBG + Gastro-jejunostomy	1	2.9%
	Open VBG + LSG + RYGB	1	2.9%

*LSG* laparoscopic sleeve gastrectomy, *RYGB* Roux-en-Y gastric bypass, *VBG* vertical banded gastroplasty

Revisional or conversion surgeries highlighted the prominence of RYGB (35.3%), SADI-s (20.6%), and OAGB (20.6%), with other methods such as banded RYGB and distalization showing lower frequencies **(**Table 3**)**.

**Table 3:** Distribution of revisional or conversion surgeries among study participants

	Types of surgeries	Count	%
Revisional or conversion surgery	RYGB	12	35.3%
	SADI-s	7	20.6%
	OAGB	7	20.6%
	Banded RYGB	6	17.6%
	Banded RYGB + Distalization	1	2.9%
	Distalization	1	2.9%

*OAGB* one anastomosis gastric bypass, *RYGB* Roux-en-Y gastric bypass, *SADI-s* single-anastomosis duodenoileal bypass with sleeve gastrectomy

The average time consumed in TBL estimation was 19 ± 4 min, ranging from 14 to 26 min. The estimated mean TBL using 3D gastric volumetry CT scan was 680 cm, with an SD of 78 cm, ranging from 565 to 885 cm. On the other hand, intraoperative TBL measurement using pre-marked graspers yielded a mean of 649 cm (SD, 121 cm; range, 460–980 cm), with no statistically significant difference between the methods (*P* = 0.264).

**Fig. 5:**
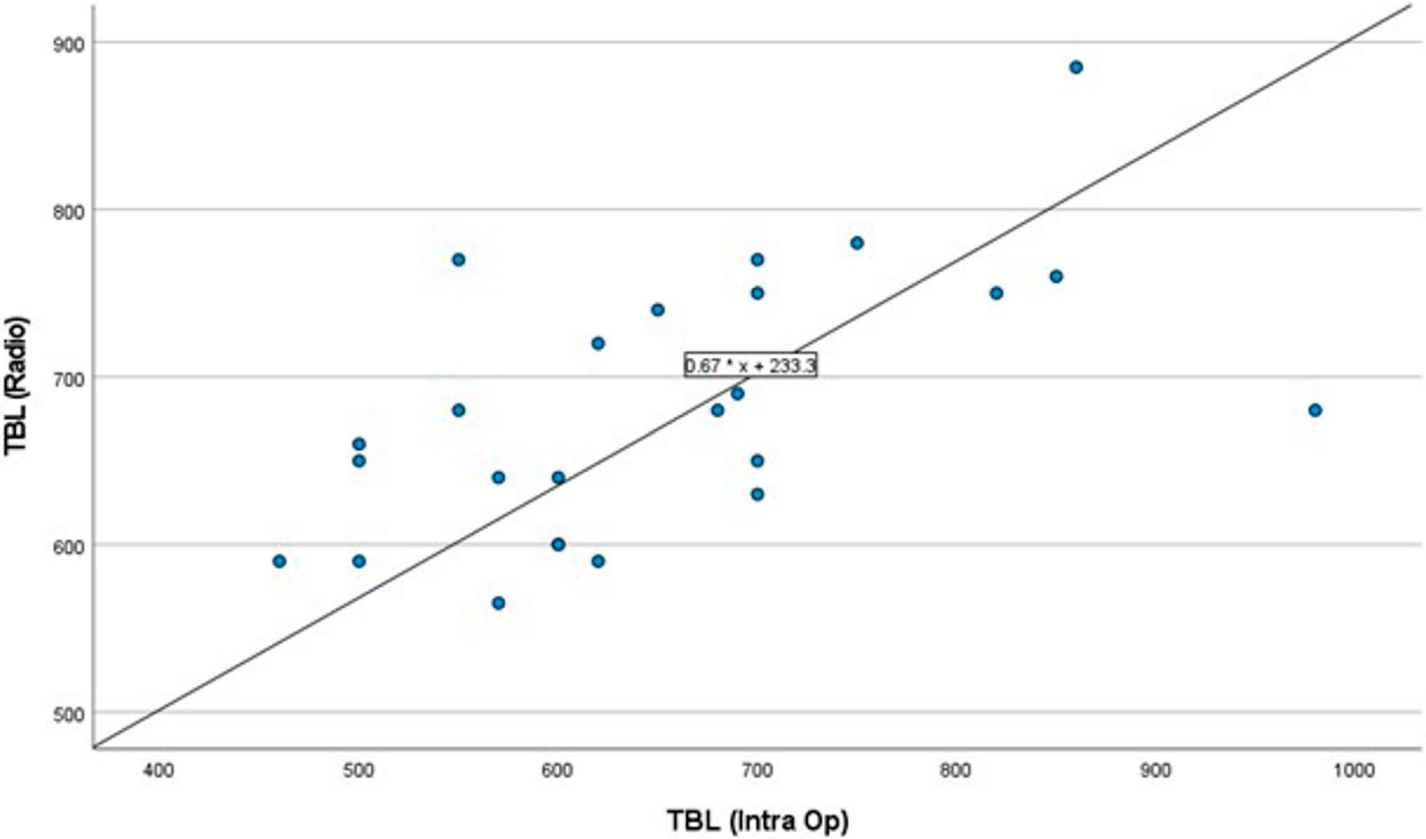
Scatter plot showing the correlation between radiographic and intraoperative total bowel length estimation among assessed patients. Op: Operative, TBL: Total Bowel Length

The intraclass correlation (ICC) for radiographic and intraoperative measurements showed that radiographic assessment of TBL had moderate reliability, with single-measure ICCs of 0.530 (*P* = 0.007) and average-measure ICCs of 0.693 (*P* = 0.007) **(**Fig. 5**).**

We analyzed the patient-by-patient readings from both techniques — i.e., intra-operative and 3D CT — and determined the percentage of variability in each case.

The results revealed the presence of variability between the two measurement techniques of < 10% in 48% of the study population, variability of 10–15% in 20% of the study population, and variability of > 15% in 32% of the study population.

A significant association was observed between the variation amount and gender (*P* 0.046). Females were to some extent more evenly distributed across the variation categories, with the largest proportion in the > 15% group (44.4%), whereas the majority of males were in the < 10% category (85.7%), and no males were in the > 15% category. No statistically significant differences were detected between variation categories with respect to BMI (*P* = 0.824), operative time (*P* = 0.431), or age (*P* = 0.730).

## Discussion

Measuring small intestinal length in the context of bariatric bypass surgery is critical for optimizing surgical outcomes and addressing complications related to nutrient absorption [3]. Accurate assessment of the small intestine can significantly influence the effectiveness of procedures such as RYGB, where the bowel length plays a pivotal role in postoperative results [10].

Surgical measurement is the traditional approach for measuring small intestinal length [9]. Recent advancements in imaging techniques, particularly 3D CT volumetry and in vivo laparoscopic measurements using pre-marked instruments, have emerged as valuable tools in this field [10].

The traditional intraoperative technique for bowel measurements performed by a surgeon is notably inconsistent. This unreliability can stem from various factors such as the degree of exposure, the tension applied during measurement, the tools utilized, and the person carrying out the assessment [19, 20]. Moreover, by the time this evaluation occurs, the choice to proceed with surgery has already been finalized, with the patient under anesthesia and initial surgical steps taken [21].

Isreb et al. investigated bowel length using instrument markings versus intraoperative visual judgment. Their findings indicated that marked laparoscopic instruments are a simple and effective method to enhance the precision of length measurements [16]. Similarly, Jackson et al. investigated using premeasured tape vs. visual judgment. They suggested that a measurement tool for laparoscopic length measurement can improve precision without affecting operative time or procedural flow in more experienced operators [17].

In a previous study, bariatric surgeons and surgical residents measured the cadaver intestine in a laparoscopic box using marked graspers [18]. The findings revealed that experienced bariatric surgeons can estimate small bowel lengths with less than 10% deviation from target lengths. However, 51% of the estimated bowel lengths deviated by more than 10%, and 30% exceeded 15% deviation from the target lengths. Additionally, surgical residents tended to inaccurately estimate small bowel lengths by systematically underestimating the lengths of the limbs [18].

These findings highlight the discrepancies and inaccuracies associated with intraoperative visual assessment of bowel length. They raised awareness among surgeons regarding the necessity of identifying a reliable alternative that could replace or supplement their visual judgment of bowel length. A study dating back to 1984 found that measurements obtained by surgical studies and investigations are comparable to anatomical observations, such as using double-contrast small bowel enemas [12].

Another research study comprising more recent modalities compared Barium follow-through (BAFT) with CT enterography (CTe) and declared that CTe is far more accurate than BaFT [9].

In a 2017 study, researchers investigated the reliability and accuracy of determining the length of the small intestine using Magnetic Resonance Enterography (MRE) in a murine model. The findings demonstrated the feasibility of implementing a dedicated, computer-vision-based algorithm specifically designed for this purpose, underscoring its potential utility in both preclinical and clinical settings for assessing gastrointestinal anatomy and abnormalities [11].

Since 3D CT volumetry has been increasingly applied in surgical planning and gastrointestinal assessments due to its non-invasive nature and the detailed anatomical visualization it provides [22], the current study employed 3D CT volumetry to assess its measurement compared to the traditional intra-operative method using laparoscopy. The present study found no significant differences between TBL (*P* = 0.007) by 3D CT and intraoperative laparoscopic judgment during bariatric surgeries. Furthermore, the results showed moderate reliability for TBL measurement using 3D CT volumetry, indicating its ability to assess bowel lengths.

Interestingly, several researchers have recently developed and validated a real-time application for laparoscopy utilizing optical 3D reconstruction via a computer-assisted bowel length measurement system (BMS) [13–15]. These studies concluded that the BMS assessed bowel lengths with improved optimal response and precision for smaller laparoscope-to-bowel distances, while accuracy decreased with increasing distances. One study in 2021 compared the accuracy of 3D CT in estimating small bowel lengths compared to traditional surgical measurements. The research indicated that this imaging modality could capture variations in bowel anatomy and provide reliable length estimates, which are crucial for planning bypass procedures, as the length of the alimentary limb affects absorption and weight-loss outcomes [13]. However, such techniques still face technical in vivo challenges, including hardware complexity, endoscopic pose, bleeding, and smoke [13–15].

The average time consumed in TBL estimation was 19 ± 4 min, ranging from 14 to 26 min. The variability in the duration of small bowel length measurement was mainly due to technical challenges, such as the presence of adhesions, since all the patients had prior bariatric procedures or pelvic surgeries, which create a medium for adhesions. Others had a very high BMI over 50 kg/m^2^, and these patients had a high percentage of visceral fat and a heavy mesentery, making bowel measurement more difficult.

It is worth noting that among the current study participants, gender was significantly associated with variation amount, with higher variation more common in females. BMI, operative time, and age showed no such association.

However, measuring TBL using preoperative 3D CT volumetry would spare surgeons this manual step, reducing operative time while still ensuring precise limb-length adjustment tailored to target postoperative weight loss, which is particularly beneficial for bariatric patients at increased anesthetic risk [23, 24].

The purpose of the study was to validate the accuracy of the 3D CT bowel length estimation in comparison to intra-operative bowel length estimation. The validity of the CT helps decrease complications from manual counting of the total bowel length, such as postoperative ileus and iatrogenic injuries. Intra-operative bowel limbs measurement using pre-marked graspers decreases the rate of variations and stretching, which in turn results in minimal effect on the outcome of the surgery.

An important consideration is the radiation exposure, cost, and tolerability of using 3D CT imaging. The radiation exposure from 3D CT is almost the same as that from conventional CT, between 7 and 15 mSv. Most patients have no problem taking the dye for the CT exam. Cost should not differ significantly from that of a conventional CT scan. However, the study’s location is noteworthy, as most bariatric practices are conducted privately and are not covered by insurance. Video-fluoroscopy swallow studies have lower radiation doses [25]; yet, CT offers the added value of bowel length measurement, gastric volume estimation, and detection of a hiatus hernia, facilitating better preoperative planning.

In our study, all participants were operated upon by an experienced bariatric surgeon using pre-marked graspers to reduce the error margin and hence decrease the rate of complications afterwards.

The idea is not to negate the need to measure the whole bowel length, but to minimize its risk.

### Limitations

One limitation of the current study is the relatively small sample size. However, this was attributed to the fact that 3D CT volumetry is a costly investigation technique. Additionally, the study is a single-center study.

## Conclusion

3D CT volumetry can be considered a reliable and safe method for TBL assessment. It aids in accurate preoperative planning, contributes to reduced operative time, and avoids the risks encountered during intraoperative bowel measurements in bariatric surgery.

The Preoperative 3D CT assessment aims to reduce operation-related morbidity by measuring bowel length without negating the importance of intraoperative measures.

## Recommendations

Further studies with larger sample sizes and longer follow-up periods are recommended to emphasize the current results and apply 3D CT volumetry not only in revisional or conversion bariatric surgeries but also in de novo cases. It is recommended that these concepts be explored in multicenter studies to include greater case heterogeneity and provide more substantial evidence.

## Supplementary Information

Below is the link to the electronic supplementary material.

Supplementary Material 1

## Data Availability

The findings of this study are supported by data that can be obtained by contacting the corresponding author. Access to the data is restricted because it contains details that may compromise the privacy of the research participants.
